# Defect Engineering in Zr (IV)- and Ti (IV)-Based Metal–Organic Frameworks to Enhance Photocatalytic Properties

**DOI:** 10.3390/molecules31071080

**Published:** 2026-03-25

**Authors:** Adan Martinez, Emily Pearce, John Kurowski, Daniel S. Kissel

**Affiliations:** Department of Chemistry, Lewis University, One University Parkway, Romeoville, IL 60446, USA; adanmartinez@lewisu.edu (A.M.); emilympearce@lewisu.edu (E.P.); johnakurowski@lewisu.edu (J.K.)

**Keywords:** MOFs, defect engineering, photocatalysis, photodegradation, modulators

## Abstract

Metal–organic frameworks (MOFs) are unique microporous materials being explored for a wide range of applications. Their porosity and high surface areas can readily be exploited for guest–host interactions, separations, and photochemical catalysis, but many suffer from poor charge separation and fast electron–hole recombination. Introducing structural defects, such as missing linkers or metal nodes, can create unsaturated metal sites and alter band structure, conductivity, and light absorption, improving photocatalytic performance. UiO-66-NH_2_ and MIL-125-NH_2_ are water-stable, visible-light-absorbing MOFs well suited for photocatalytic degradation of organic dyes. In this work, the influence of defect engineering on photocatalytic properties of MOFs was investigated using formic and acetic acid modulators with UiO-66-NH_2_ and variable temperature with MIL-125-NH_2_ during synthesis. The resulting materials were characterized by XRD, FTIR and SEM/EDS. Defect states were tracked using N_2_ adsorption/BET analysis and UV–Vis spectroscopy. Photocatalytic activity was evaluated by monitoring Rhodamine B (RhB) degradation in aqueous solution under simulated solar irradiation. It was found that increased temperature beyond 120 °C during synthesis promotes mesopore formation and decreases the bandgap in MIL-125-NH_2_, resulting in a more photoactive material. Defective MIL-125-NH_2_ synthesized at 150 °C showed the most defects and proved to be the best photocatalyst investigated in this study. Formic acid modulation in UiO-66-NH_2_ generated smaller crystallites that slightly increased the bandgap; however, the surface area decreased proportionally with the amount of formic acid used. The decreased surface area and observed enhancement in photocatalytic degradation of RhB suggest that formic acid introduces defects into the UiO-66-NH_2_ framework that enhance photocatalytic properties. UiO-66-NH_2_ treated with acetic acid resulted in larger crystals, increased bandgaps, and increased surface areas, suggesting that acetic acid simply modulates growth rather than imparting defects to the framework.

## 1. Introduction

Metal–organic frameworks (MOFs) are currently being explored for a wide range of applications, including chemical separations and catalysis, due to their unique porosity and large surface areas. MOFs are made of multi-dentate organic molecules, or linkers, bridging metal ion clusters. Porous materials like MOFs have large surface areas that have been shown to enhance heterogeneous catalysis through proximity effects, where more reactant molecules absorb in closer proximity, thereby facilitating more reactions [1,2,3,4,5,6]. In addition, certain MOFs with semi-conductive bandgaps perform well as photocatalysts and have shown the ability to photocatalyze different reactions, including the Hydrogen Evolution Reaction (HER), CO_2_ reduction, and degradation of organics dyes and insecticides [7,8]. Unfortunately, like many other semi-conductors, MOF photocatalysts are plagued by issues related to high activation energies and low electron–hole efficiencies due to fast recombination rates [9]. In order to combat these issues, MOFs can be modified in different ways to enhance their photoreactive properties [10,11].

**Figure 1:**
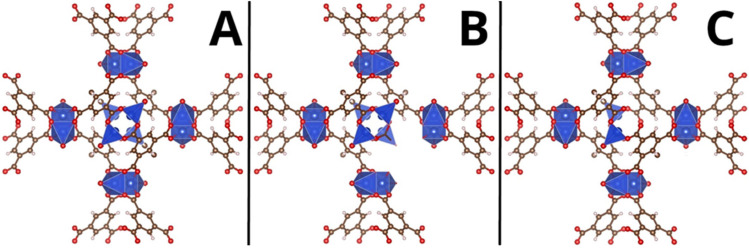
Potential defects in Metal-Organic Frameworks. The metal clusters are shown in blue and the organic linkers are represented by the other atoms. (**A**) Shows a defect-free lattice, (**B**) Shows a missing linker defect, and (**C**) shows a missing metal cluster defect.

The introduction of structural defects into the MOF framework is one unique way to impart modifications in a simple and cost-effective manner (Figure 1). Structural defects in the form of missing clusters and linkers have the ability to open the framework and increase available reaction sites, which greatly improves catalytic ability [12,13]. Missing linkers create additional void spaces and coordination vacancies on two neighboring metal nodes, and missing metal clusters leave additional void spaces and uncoordinated groups on neighboring linker molecules [14,15,16]. All MOFs are susceptible to these types of structural defects, whether intentional or unintentional, which can alter stability, adsorption, and photocatalytic properties in different ways [17,18,19]. By carefully controlling the introduction of defects through defect engineering, MOFs can be tuned for better performance in a desired application [20,21]. MOFs with a greater number of defects have shown an increased ability to adsorb and activate reactant molecules, improving overall catalysis. For instance, the Zn(II)-based MOF Zn-TMU was found to absorb more CO_2_ after a solvent-assisted linker exchange, which was used to induce missing linker defects, compared to its pristine counterpart [22]. Defective MIL-125-NH_2_ produces hydrogen gas at a rate 3.5 times faster than pristine MIL-125-NH_2_ [23]. Additional studies have also shown that defective MOFs are significantly more efficient at catalyzing CO_2_ reduction than defect-free MOFs [24,25].

Defect states can be engineered in MOFs during synthesis by incorporating modulators or by subjecting the MOF to different conditions, such as varying heat and pH [26,27]. Modulators are mono-dentate molecules or ions that compete against the multi-dentate linker for metal coordination. The addition of a modulator during synthesis can impact the rate at which the crystal structure forms, altering crystal size and morphology [28]. When compared to a standard synthesis, UiO-66 synthesized with the presence of a modulator was seen to have larger, more homogeneous octahedral crystals [29]. The increase in MOF particle size in the presence of a modulator is due to competition between the modulator and linker, which can cause nucleation and growth rate to slow while crystal size increases [29,30,31]. Missing linker sites can result from the modulator remaining attached to the secondary binding unit, increasing the amount of unsaturated metal centers [28]. Exposing additional metal sites alters the ability to transfer electrons, which in some instances can improve catalytic properties [12,22].

In addition, altering the temperature at which synthesis occurs can alter the number of defect sites present. An increase in synthesis temperature has also been correlated with smaller particle size, as an increase in temperature can result in a faster nucleation step [12,31]. Through altering the temperature at which synthesis occurs, vacancies within the framework can arise from atoms coordinating in different positions or being removed from the crystal lattice altogether. These framework vacancies can alter the band structure and electronic properties of the material, which can influence reaction efficiency [32]. When UiO-66-NH_2_ was synthesized at a range of temperatures, it was found that the number of defects increased with increasing temperature [33]. This trend was verified using powder X-ray diffraction (XRD), which showed that the low-angle peak associated with the presence of defects became more intense as synthesis temperature increased [24].

The investigation herein explores defect engineering in the MOFs UiO-66-NH_2_ and MIL-125-NH_2_. UiO-66-NH_2_ is a zirconium-based MOF that is highly stable in aqueous solution, making it an attractive material for long-term use in water remediation [34]. Its remarkable stability has been attributed to its high degree of connection, as each zirconium metal is connected to twelve different 2-aminobenzene-1,4-dicarboxylate linkers [10]. However, the highly connected metal oxide nodes are insulating by nature. MIL-125-NH_2_ is a photoactive titanium-based MOF that features low toxicity and moderate solution durability [23]. UiO-66-NH_2_ was synthesized with varying concentrations of acetic and formic acids, while MiL-125-NH_2_ was synthesized at different temperatures in order to introduce defects. Each MOF was characterized using infrared spectroscopy, bandgap analysis, surface area and pore volume analysis, and photodegradation studies in both pristine and defect-induced products. Surface area is particularly important when characterizing the influence of defects as it directly relates to the structure and stability of the framework [35]. While a change in surface area and pore volume indicates defect states introduced into the framework, too many defects can also lead to instability and framework collapse.

## 2. Results and Discussion

### 2.1. PXRD Characterization

**Figure 2:**
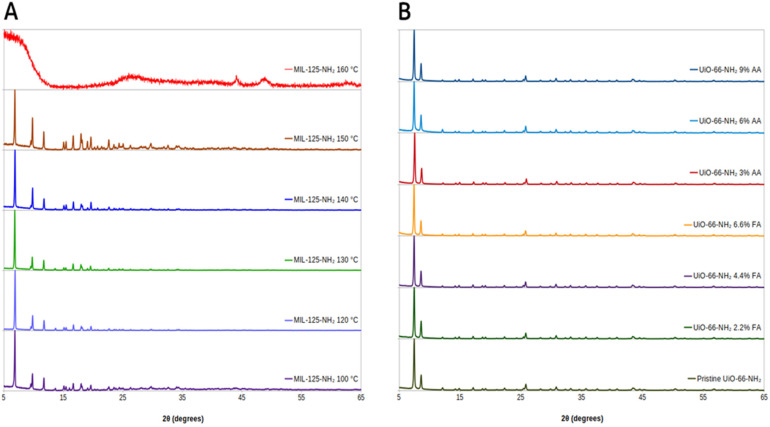
PXRD spectra of (**A**) MIL-125-NH_2_ and (**B**) UiO-66-NH_2_.

PXRD spectra for MIL-125-NH_2_ MOFs can be seen in Figure 2A. All MIL-125-NH_2_ MOFs up to a 150 °C synthesis temperature show the strong diffraction peaks at 2θ = 6.9°, 9.8° and 11.7° that are characteristic of the MIL-125-NH_2_ lattice and indicate successful crystal formation. Secondary peaks at 2θ = 16.6°, 18°, 19.6°, 22.6°, 23.8° and 24.3° become more prominent as synthesis temperature increases or decreases from 120 °C. This enhancement in relative intensity, along with increased noise at low 2θ, corresponds to more prominent secondary phases as accumulated defects distort the crystal lattice. This is further supported by the changes in surface area and pore volume shown in the defect analysis below. MIL-125-NH_2_ synthesized at 160 °C shows highly amorphous character and poor crystallinity.

PXRD spectra for UiO-66-NH_2_ MOFs can be seen in Figure 2B. The characteristic diffraction peaks at 2θ = 7.5° and 2θ = 8.5° indicate successful crystal formation even with modulator addition. There is no significant peak broadening or shifts, nor any increases in secondary peaks on any sample compared to pristine UiO-66-NH_2_. If the use of modulators indeed altered the MOF crystal, their impact was not enough to change the long-range order of the MOF lattice, indicating a different kind of defect from chemical modulation compared to temperature modulation of MIL-125-NH_2_.

### 2.2. FTIR Characterization

**Figure 3:**
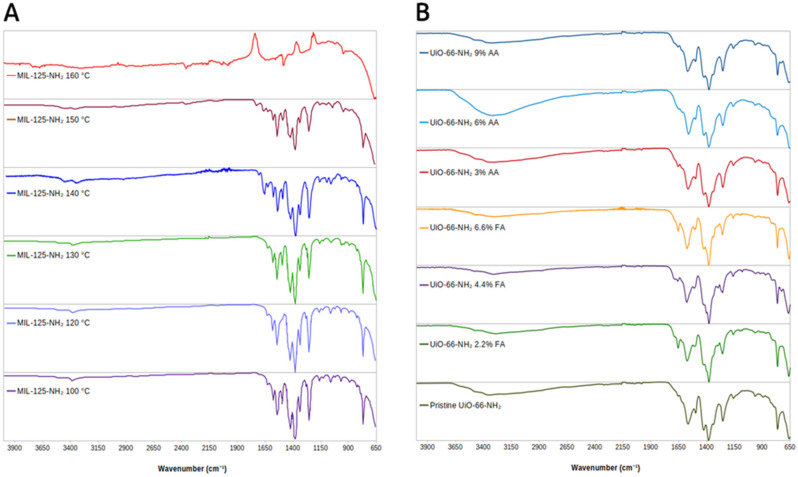
FTIR spectra of (**A**) MIL-125-NH_2_ and (**B**) UiO-66-NH_2_.

FTIR spectra for MIL-125-NH_2_ MOFs can be seen in Figure 3A. The fingerprint region shows peaks consistent with those reported for pristine MIL-125-NH2 except for the MOF synthesized at 160 °C. The sharp peak at 765 cm^−1^ is due to the O-Ti-O stretching frequency of the metal cluster. The peak at 1260 cm^−1^ is due to the C-N bond from the amino nitrogen bound to the BDC linker. The intense peaks at 1380 cm^−1^ 1530 cm^−1^ are due to symmetric and asymmetric stretches of coordinated COO− groups. The two peaks at approximately 3200 and 3300 cm^−1^ arise from the NH2 group of the linker. When MIL-125-NH2 is synthesized at 140 and 150 °C, there are additional shoulder peaks that show up between 1650 and 1700 cm^−1^ from protonated COOH groups that result from defects introduced in the form of missing metal nodes. When COO− is no longer coordinated to the metal center and becomes protonated, the asymmetric stretching frequency shifts to higher wave numbers. The loss of product peaks in the MIL-125-NH2 synthesized at 160 °C is indicative of an unsuccessful synthesis and/or framework collapse.

FTIR spectra for UiO-66-NH_2_ MOFs can be seen in Figure 3B. The sharp peak at 765 cm^−1^ is due to the O-Zr-O stretch of the metal cluster. The peaks observed at approximately 1390 and 1580 cm^−1^ are characteristic of symmetric and asymmetric stretching frequencies of the coordinated carboxylate groups from the BDC-NH_2_ linker. The C-C stretches of the aromatic ring of the BDC-NH_2_ linker give rise to frequencies near 1490 cm^−1^ and 1430 cm^−1^. The broad peak between 3200 and 3400 cm^−1^ is characteristic of the amine groups of the BDC-NH_2_ linkers. The UiO MOFs treated with AA show differences in the relative intensities of the N-H stretching frequencies between 3200 and 3400 cm^−1^ when compared to pristine UiO-66-NH_2_. These changes suggest protonation of the amino group. The UiO-66-NH_2_ MOFs treated with FA and 3% AA display a characteristic shoulder peak at 1650 cm^−1^, a property of carboxylate groups, due to FA being trapped in the pores or bound to the metal clusters. This indicates a stronger interaction of FA compared to AA, where FA is more liable to stay bound inside the MOF, likely due to its smaller size.

### 2.3. Defect Analysis

The internal surface area and porosity of each MOF were determined from the nitrogen adsorption isotherms. The BET and T-plot methods were used to determine surface area and micropore size, which can be observed in Supplementary Table S1. The surface area and pore sizes of MIL-125-NH_2_ synthesized at 120 °C are taken as a reference, since this sample has the largest surface area. UiO-66-NH_2_ synthesized with no modulators (pristine) is also taken as a reference. The difference in surface area and pore size of each defective MOF relative to the pristine reference was calculated using Equations (1) and (2) below, where *SA* stands for surface area and *PV* stands for pore volume.

(1) $${SA}_{RD}=\frac{{SA}_{Sample}-{SA}_{Reference}}{{SA}_{Reference}}$$

(2) $${PV}_{RD}=\frac{{PV}_{Sample}-{PV}_{Reference}}{{PV}_{Reference}}$$

Equations (1) and (2) provide the relative difference between surface area (*SA_RD_*) and pore volume (*PV_RD_*) parameters.

**Figure 4:**
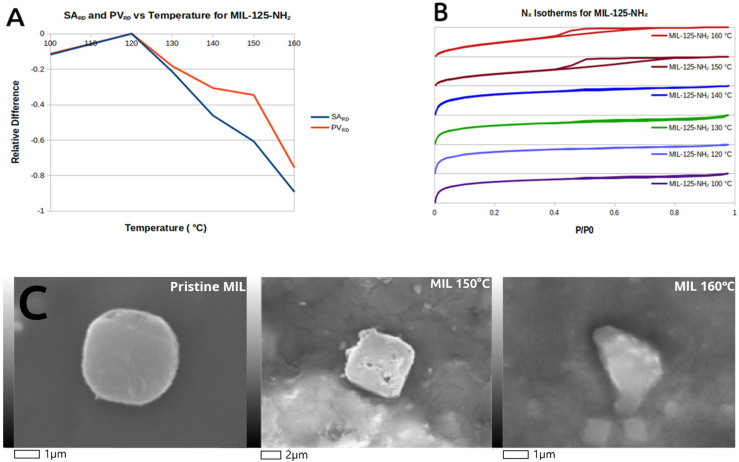
Qualification of MIL-125-NH_2_ defects. (**A**) SA_RD_ and PV_RD_ vs. temperature. (**B**) N_2_ isotherms. (**C**) SEM images of MIL-125-NH_2_ synthesized at 120, 150 and 160 °C.

The *SA_RD_* parameter for MIL-125-NH_2_ steadily and sharply decreases with temperature in an almost linear fashion. Temperature modulation in principle would allow for missing linkers and metal node defects to form in the lattice, but these defects are large enough to partially collapse the MOF lattice of MIL. This is evidenced by the N_2_ isotherm, showing increasing hysteresis with increased synthesis temperature (Figure 4B). Hysteresis is more characteristic of mesoporous materials, and not of the microporous MIL-125-NH_2_, thus making temperature-modulated defects macroscopic and of the cavitation type, but these are not arbitrarily large. The decrease in the PV_RD_ parameter plateaus between 140 and 150 °C, indicating that pore volume decreases up to a point or critical size. In the 140–150 °C range, cavitations do not become larger but they become more abundant. This correlates with the increased secondary peaks in the powder XRD. Additionally, these cavitations can be observed in the SEM/EDS for MIL-125-NH_2_ synthesized at 150 °C. The faces of the crystal are dotted with cracks and holes compared to pristine MOF. The crystal shape is also significantly changed from a pristine semicircular wafer to a square-like structure. Despite this, the material still retains MOF-like adsorption, evidenced by moderate gas uptake at low pressures in the N_2_ isotherms.

At 160 °C, these defects accumulate enough to completely overtake the long-range order of the MOF lattice and form a completely amorphous material. It might be possible to synthesize an even more defective MOF at this temperature by decreasing synthesis time, if defects form after MIL-125-NH_2_ crystallization.

SA_RD_ and PV_RD_ parameters are correlated in UiO-66-NH_2_ for both FA and AA, meaning that surface area is directly correlated with pore volume in these MOFs. Interestingly, the behavior for both modulators is opposite. For FA, SA_RD_ increases at low modulator concentration and then decreases, while AA decreases SA_RD_ at low modulator concentration but later increases at higher concentrations.

**Figure 5:**
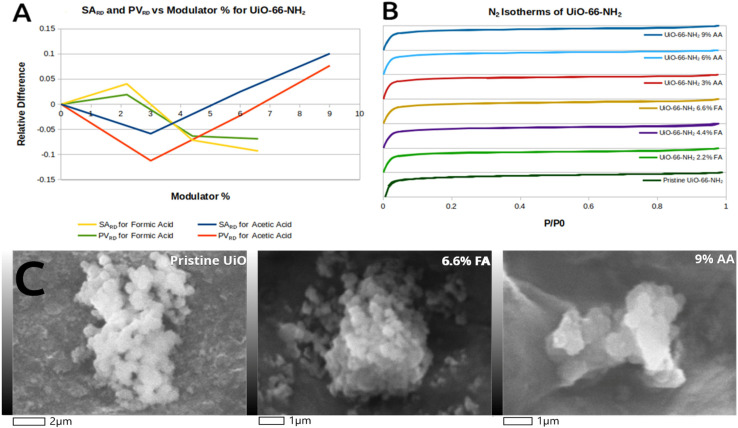
Qualification of UiO-66-NH_2_ defects. (**A**) SA_RD_ and PV_RD_ vs. modulator Vol%. (**B**) N_2_ isotherms. (**C**) SEM images of UiO-66-NH_2_ with modulators at highest modulator concentration.

FA is a strong coordination competitor of BDC-NH_2_, even at low concentrations, and can be captured in the lattice via metal coordination at the metal clusters or hydrogen bonding to the functional groups of the BDC-NH_2_ linker. This is evidenced by the weak IR peak at 1650 cm^−1^ present in all FA-modulated UiO-66-NH_2_ products (Figure 3B). Higher concentrations of FA effectively modulate crystal growth, producing smaller crystals (Figure 5C), and introduce defect states that decrease SA_RD_. These defects likely arise from missing metal clusters within the framework. A previous study from Shearer et al. showed that the strength of an acid modulator is directly proportional to the number of metal cluster defects in UiO MOFs [15]. Formic acid is the strongest acid modulator used in this study, with a pK_a_ = 3.75.

While AA features the same functional groups as FA, it is a weaker acid (pK_a_ = 4.76 vs. 3.75) and larger in size than FA, making it a weaker coordination competitor and less likely to remain trapped in the framework. Previous reports using AA modulators with Zr-based MOFs have shown similar behavior, with surface area correlating directly with concentration of the modulator [36,37]. These studies also reported increases in pore volume and surface area with modulator concentration except at the extreme highest concentrations, where degradation occurs. The AA-modulated UiO-66-NH_2_ product reported here follows this trend of increasing SA_RD_ and PV_RD_ with increasing AA concentration except for treatment with 3% AA. The initial decrease in SA_RD_ and PV_RD_ for 3% AA is attributed to small amounts of AA trapped in the UiO-66-NH_2_ framework, which is evidenced by a small shoulder peak around 1650 cm^−1^ in the IR (Figure 3B). The increase in surface area at higher AA concentrations is likely the result of missing linker defects introduced by AA modulation during framework formation. A previous report by Piciorus et al. showed that AA-modulated UiO MOFs have larger surface areas as a result of decreases in the number of linkers connected to metal nodes [36].

**Figure 6:**
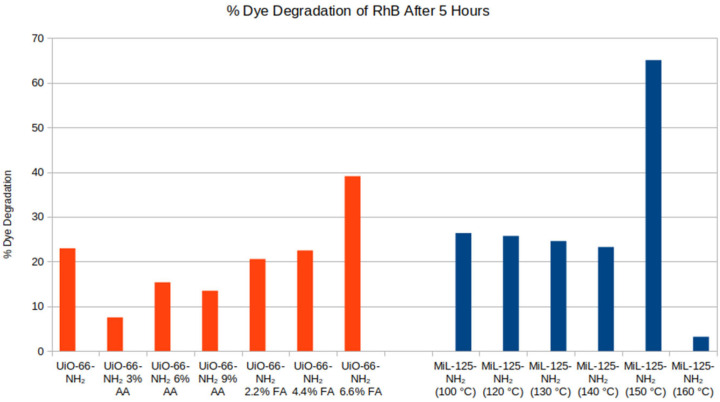
Percent dye degradation after 5 h for each MOF sample.

Overall, the relative differences in surface area and pore volume are minimal (<0.1) compared to pristine UiO-66-NH_2_, even for the highest modulator concentrations. The influence of acid modulation on the MOF framework appears relatively “mild” for UiO MOFs, as SEM/EDS images reveal no significant differences in crystal morphologies and adsorption/desorption curves show no hysteresis present in any FA- or AA-modulated products (Figure 6). The FA-treated MOFs show smaller crystal sizes, whereas the AA-treated MOFs show little change in crystal size.

### 2.4. Bandgap Analysis

Bandgap energies were calculated using the method outlined by P. Makuła et al. [11]. This was done by applying the Kubelka–Munk function using reflectance spectra and fitting the data according to the Tauc method, assuming an infinitely thick sample. Briefly, the Kubelka–Munk function was graphed against photon energy and the bandgap was determined by extrapolating the most linear portion of the band edge. Plots of the Kubelka–Munk function vs. photon energy for all samples are shown in Supplementary Figures S3 and S4. The bandgaps determined from these plots are listed below in Table 1.

Bandgap measurements for acid-treated UiO-66-NH_2_ MOFs slightly increased compared with pristine UiO-66-NH_2_; however, the type of modulator used and concentration did not further influence the bandgap energy. The measured bandgaps show no variation between formic and acetic acid treatments and do not vary with increasing formic and acetic acid concentration. This indicates that treatment with formic and acetic acid does not appreciably change the bandgap of UiO-66-NH_2_ within the concentrations used in this study.

The bandgap measurements for MIL-125-NH_2_, however, show appreciable changes with temperature variation. As the temperature during synthesis is increased beyond 130 °C, the bandgap decreases. Uncoordinated metal ions and free carboxylate groups give different hybridizations, altering the local crystal fields. These defects introduce new frontier orbitals that effectively lower the bandgap [38]. Additionally, uncoordinated BDC-NH_2_ absorbs light differently, raising the valence band edge and shrinking the bandgap further [38].

### 2.5. Photodegradation

Photodegradation studies were conducted on all MOFs to determine how defect states influenced photocatalytic properties. Solutions containing UiO-66-NH_2_ MOFs in Rhodamine B (RhB) were monitored over 28 h. Absorbance measurements were taken at 20 min, 40 min, 1 h, 1.5 h, 2 h, 2.5 h, 3 h, 4 h, and 5 h, and then the solution was left to be stirred under continuous light irradiation overnight. Absorbance measurements resumed the next day at 24, 25, 26, 27, and 28 h. Plots showing normalized absorbance versus time are given in Supplementary Figures S5 and S6. All UiO-66-NH_2_ MOFs were able to photodegrade RhB; however, the rate of degradation was slow, and some MOFs only degraded 30–40% of RhB over the entire 28 h. Additionally, the UiO-66-NH_2_ MOFs treated with AA did not show enhanced RhB photodegradation relative to pristine UiO-66-NH_2_. The only MOF that performed better than pristine UiO-66-NH_2_ for RhB photodegradation was the UiO-66-NH_2_ treated with 6.6% FA, which was the highest concentration of FA used in this study.

The MIL-125-NH_2_ MOFs performed better at photodegrading RhB, with all MOFs, except the MIL-125-NH_2_ synthesized at 160 °C, reaching 100% RhB remediation over the 28 h period investigated; however, the rate at which RhB was photocatalytically reduced varied. Plots of normalized absorbance of RhB over 5 h of light irradiation in the presence of each MIL-125-NH_2_ MOF are provided in Supplementary Figure S6. The MIL-125-NH_2_ MOF synthesized at 150 °C performed best as a photocatalyst, degrading approximately 65% of RhB in only 5 h. The MIL-125-NH_2_ MOFs synthesized at 100, 120, 130 and 140 °C only remediated ~25% of RhB over the same time period. MIL-125-NH_2_ synthesized at 160 °C showed no RhB removal, which is likely a result of framework collapse or incomplete framework formation producing a material with altered photoelectronic properties. A comparison of percent RhB removal over 5 h of light irradiation for all the MOFs tested in this study (MIL-125-NH_2_ and UiO-66-NH_2_) is given in Figure 6.

**Figure 7:**
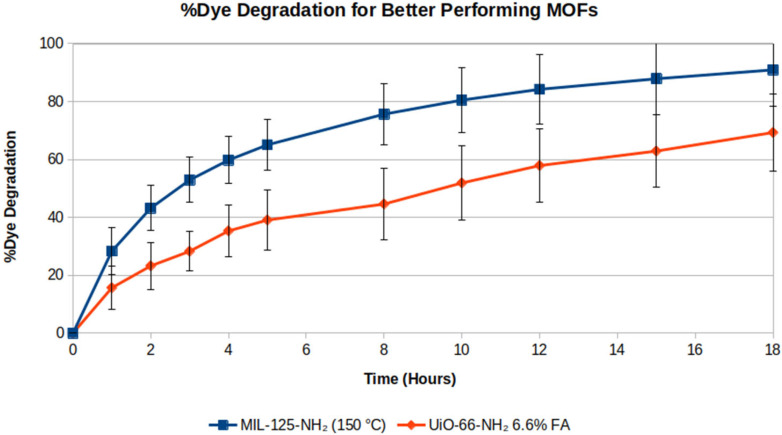
Percent dye removal of MIL-125-NH_2_ (150 °C) and UiO-66-NH_2_ (6.6% FA) over 18 h.

Overall, the UiO-66-NH_2_ treated with 6.6% FA and MIL-125-NH_2_ synthesized at 150 °C performed best at photocatalytically degrading RhB in solution rapidly. A comparison of both materials was done by running photodegradation experiments over 18 h to evaluate the efficiency and performance of each photocatalyst relative to the others. Figure 7 shows results for % RhB dye removal over time for each material.

MIL-125-NH_2_ synthesized at 150 °C degrades nearly 100% of RhB dye in 18 h, whereas UiO-125-NH_2_ treated with 6.6% FA reaches roughly 70% degradation over the same period. The enhanced photocatalysis observed in MIL-125-NH_2_ 150 °C is likely a bandgap-driven phenomenon. MIL-125-NH_2_ 150 °C had the smallest bandgap (2.39 eV) of all the viable MOF products investigated, making it better at harvesting light and generating reactive species in solution necessary for RhB reduction.

Similar results have been obtained by Ngan Tran et al. in the degradation of Rhodamine B by bimetallic MOFs [39]. Their study utilized similar synthesis and testing procedures with particular emphasis on their solvothermal synthesis of MOFs at 150 °C. Bimetallic FeCo MOFs are able to degrade 90% of RhB in about 2 h of light irradiation, whereas the defective MIL-125-NH_2_ 150 °C takes about 15 h. While defect states were not a focus of their study, the low surface area and high signal-to-noise ratio of their PXRD suggest that defect states played a major role in the fast degradation rate. It should also be noted that these FeCo MOFs have very close bandgap values to MIL-125-NH_2_ 150 °C (2.32 vs. 2.39 eV), highlighting defect engineering as an effective bandgap modulator.

### 2.6. Adsorption Capacity and Adsorption Kinetics

**Figure 8:**
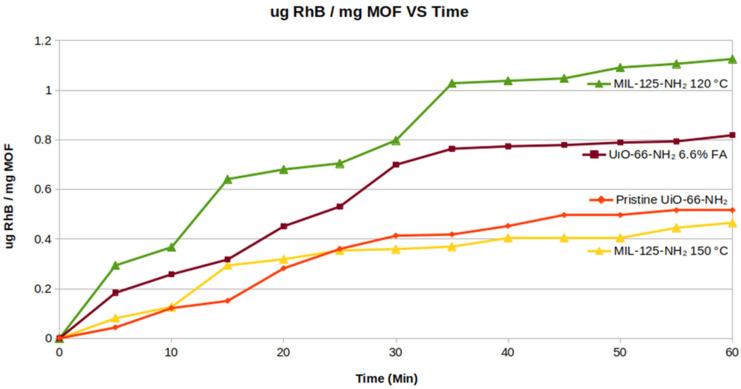
Adsorption of Rhodamine B on tested MOFs over time.

To differentiate physical dye adsorption from photocatalytic degradation, adsorption capacity experiments were conducted on pristine UiO-66-NH_2_, UiO-66-NH_2_ 6.6% FA, MIL-125-NH_2_ 120 °C, and MIL-125-NH_2_ 150 °C. Each material was left to soak up dye solution in the dark until physical adsorption of RhB reached an equilibrium state (~1 h) to determine overall adsorption capacity at equilibrium. Figure 8 shows results for adsorption capacity in mg of RhB per mg of MOF as a function of time. These results correlate well with surface area and pore volume (Table 2), showing that MOFs with larger surface areas and pore volumes had higher adsorption capacities for RhB dye.

MIL-125-NH_2_ 120 °C displays the highest adsorption capacity for RhB by virtue of having the highest surface area and largest pore volumes, and MIL-125-NH_2_ 150 °C adsorbs the least amount of RhB as it has the lowest surface area and smallest pore volume. UiO-66-NH_2_ 6.6% FA adsorbs more dye than its pristine counterpart despite having slightly smaller surface area and lower pore volumes. This difference is likely due to the nature of the FA modulator treatment, considering that the surface area and pore volume do not show large differences (as opposed to the MIL-125 MOFs). The introduction of metal node defects through FA modulation likely alters the surface charge characteristics of the MOF, which influences the electrostatic attraction of RhB to the MOF surface, creating a slight difference in adsorption capacity.

**Figure 9:**
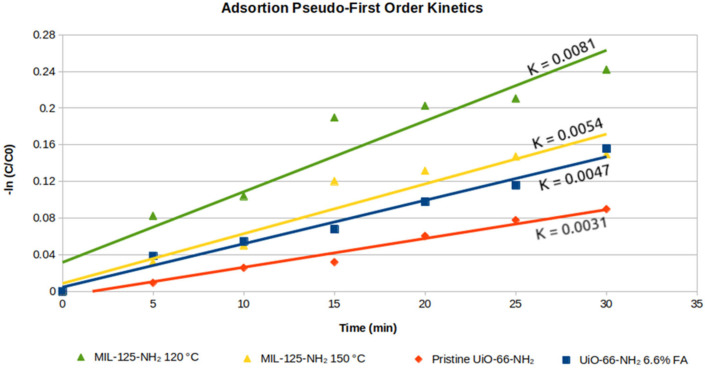
Adsorption kinetics of MOFs.

The RhB adsorption data was fit to different kinetic models to determine the rate of adsorption. The data fit best to a pseudo-first-order kinetic model for all MOFs investigated. The resulting R^2^ values for all kinetic models investigated are provided in Table S2 in Supplementary Materials. The results from the pseudo-first-order kinetic plots for all MOFs investigated are shown in Figure 9.

MIL-125-NH_2_ 120 °C showed the highest rate of RhB adsorption, which is driven by its high surface area and larger pore volume. MIL-125-NH_2_ 150 °C showed the second fastest adsorption rate, most likely due to its high number of defects and macro-sized pores that allow better diffusion into the lattice. UiO-66-NH_2_ 6.6% FA is a faster absorber than its pristine counterpart, again due to its higher number of defects.

### 2.7. Scavenger Experiments

**Figure 10:**
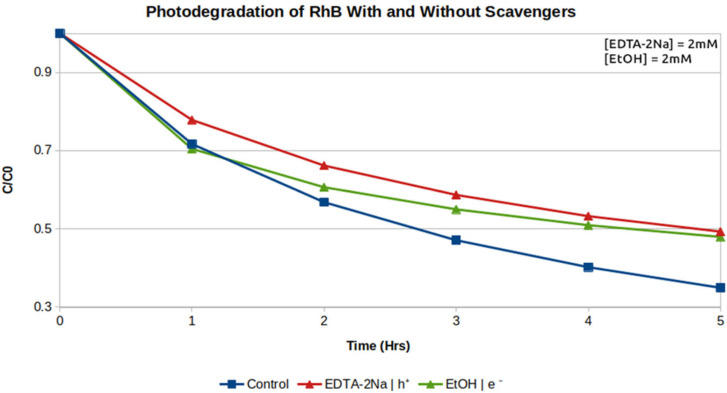
Photodegradation of RhB by MIL-125-NH_2_ with and without scavengers.

To investigate the role of the MOF photocatalyst in RhB degradation, photodegradation experiments were rerun with electron and hole scavengers present in solution. EDTA-Na_2_ was used as a selective hole scavenger and ethanol as an electron scavenger, according to previous reports of similar investigations with MIL-125-NH_2_ [40]. The results for normalized concentration of RhB over time for each scavenger experiment relative to the control are presented in Figure 10.

Experiments with EDTA-Na_2_ and ethanol in solution both showed decreases in the rate of photodegradation compared to the MIL-125-NH_2_ 150 °C control. Interestingly, addition of hole and electron scavengers in equal concentrations depresses photodegradation by the same amount after 5 h. This suggests that the MIL-125 150 °C photocatalyst is facilitating a dual hole–electron mechanism. In solution, holes generated by the photocatalyst oxidize water molecules to form hydroxide radicals and electrons in the conduction band react with dissolved O_2_ to form O_2_**^·−^** species [40]. In short, the MOF photocatalyst functions as a multi-ROS generator to indirectly degrade RhB in solution.

**Figure 11:**
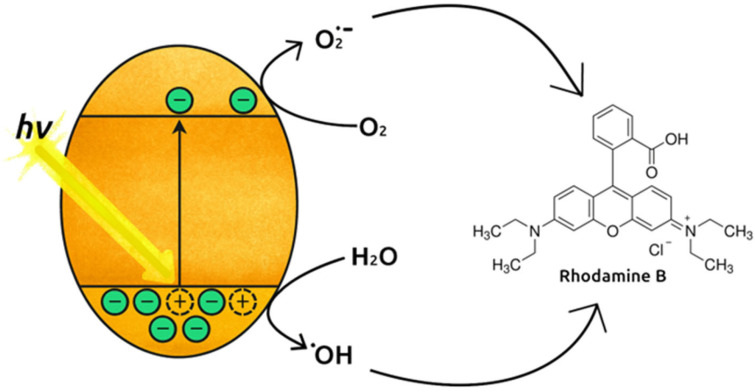
Mechanism of photogenerated ROS attack on Rhodamine B.

An illustration showing the proposed mechanism and role of the photocatalyst is provided in Figure 11. When light irradiates MOFs, it excites an electron from the valence band to the conduction band, leaving behind a hole. The photogenerated charges react with solution components to produce reactive oxygen species that later diffuse and degrade RhB absorbed to the framework.

### 2.8. Crystallinity Test

A comparison of percent crystallinity before and after photodegradation experiments was used to determine the stability of each MOF photocatalyst in solution (Table 3). This was accomplished by conducting PXRD of each MOF before and after 24 h of irradiation in dye solution. Percent crystallinity was assessed using the method of peak integration as described by the University of Utah’s Material Characterization Lab (Equation (3)) [41]. In short, the area under all diffraction peaks was summed and then divided by the total area under the entire PXRD spectra. This was done using Profex 5.6.1.

(3) $$\%Crystallinity=\frac{areaundercrystallinepeaks}{areaunderallpeaks}\times 100$$

Equation (3): %Crystallinity calculation from PXRD data.

**Table 3:** Crystallinity of MOFs after 24 h of irradiation.

MOF	% Crystallinity (0 h)	% Crystallinity (24 h)	Percent Difference	Relative Loss (C/C_24h_ × 100)
MIL-125-NH_2_ 120 °C	33.55%	31.44%	2.11%	6.28
MIL-125-NH_2_ 150 °C	5.86%	5.52%	0.34%	5.8
Pristine UiO-66-NH_2_	30.19%	29.73%	0.46%	1.52
UiO-66-NH_2_ 6.6% FA	31.23%	25.22%	6.01%	19.24

The relative loss in crystallinity sustained by MIL-125-NH_2_ after 24 h is similar for both the 120 °C and 150 °C syntheses, indicating that the MIL lattice is resilient to the 24 h photodegradation in solution despite the presence of major defect states in the 150 °C synthesis. UiO-66-NH_2_ 6.6% FA, while being a decent photocatalyst, sustains four times as much loss in crystallinity in the same time window as MIL-125-NH_2_ 150 °C, and ten times more than its pristine version. These results indicate that MIL MOFs display a major resilience to photodegradation, even in their most defective states, compared to UiO MOFs treated with acid modulators.

## 3. Materials and Methods

### 3.1. Synthesis of UiO-66-NH_2_ MOFs

Pristine UiO-66-NH_2_ was synthesized using an equimolar ratio of ZrCl_4_ and 2-aminobenze-1,4-dicarboxylic acid (BDC-NH_2_) in N,N-dimethylformamide (DMF) and HCl. The solution was heated at 120 °C for 24 h in a solvothermal reactor. The resulting product was collected using vacuum filtration, washed with DMF and activated with methanol before being dried at 60 °C for 24 h. The UiO-66-NH_2_ powder was stored in an amber vial in a vacuum desiccator until use. Defective UiO-66-NH_2_ was synthesized using the procedure described previously with the addition of a modulator during the initial heating cycle. In this instance, acetic acid (AA) and formic acid (FA) were chosen as modulators. Varying concentrations of AA (3%, 6%, and 9% by volume) and FA (2.2%, 4.4%, and 6.6% by volume) were introduced during the initial heating cycle into the solvothermal reactor to limit the rate of crystal formation. The resulting defective products are referred to as %AA UiO-66-NH_2_ or %FA UiO-66-NH_2_ based on the % volume of modulator used.

### 3.2. Synthesis of MIL-125-NH_2_ MOFs

Pristine MIL-125-NH_2_ was synthesized using a 2:1 molar ratio of 2-aminobenze-1,4-dicarboxylic acid and titanium isopropoxide in DMF and methanol. The reaction mixture was sonicated until homogeneous before being heated at 120 °C for 24 h in a solvothermal reactor. The resulting MOF product was collected via vacuum filtration and washed with DMF before being activated in methanol. The MIL-125-NH_2_ product was dried at 60 °C for 24 h, and the resulting powder was stored in an amber vial in a vacuum desiccator until use. Defective MIL-125-NH_2_ was synthesized using the same synthetic procedure but at different temperatures. Defect states were introduced into the framework by heating the reactant mixture at 130 °C, 140 °C, 150 °C, and 160 °C. The resulting defective products are referred to as MIL-125-NH_2_ (temp), where the temperature is given in parentheses.

Infrared spectroscopy (IR) was used for characterization of MOF products to determine functional groups present using a Cary 630 FTIR instrument (Agilent Technologies, Santa Clara, CA, USA) and Agilent Microlab software 9.16.00.0 (Agilent Technologies). The crystal surface of the instrument was cleaned using an acetone-soaked cotton ball before a background measurement was taken to get a baseline profile. Crystal was cleaned with acetone in between samples.

Powder X-ray diffraction (PXRD) was conducted using an ARL X’TRA Companion X-ray Diffractometer with a 600 W Cu source (Thermo Fisher Scientific, Waltham, MA, USA). The instrument was set up for a continuous 5 min scan per sample with continuous rotation.

Scanning electron microcopy (SEM) analysis was done using a benchtop Jeol JCM 7000 (JEOL Ltd., Tokyo, Japan). Samples were prepared by placing 10 mg of MOF in a solution of methanol before sonicating for 15 min. Approximately 1 mL of this solution was further diluted with methanol and sonicated for an additional 15 min. Then, 250 µL of this solution was pipetted onto a strip of copper tape before being dried at 90 °C for 10 min or until all methanol was evaporated. The samples were gold-sputtered for 30 s before being placed in the SEM for observation. Energy-dispersive spectroscopy (EDS) analysis was also performed on each sample.

The bandgap was determined through analysis of Direct Reflectance UV–Visible Spectroscopy on a Persee Analytics T8DS UV Spectrometer (Persee Analytics, Beijing, China). Powder MOF samples were packed into a sample holder with barium sulfate to create a smooth surface that reduced reflectance noise. The instrument was calibrated using a dark and light standard and a background was taken using packed barium sulfate before each measurement.

Micropore volume was measured using a NOVAtouch^TM^ WINtouch^TM^ series surface area analyzer (Quantachrome Instruments, Boynton Beach, FL, USA). The mass of the cell was recorded before adding the powder sample and taking the mass again. The mass of the sample cell was taken before and after degassing. Samples underwent degassing at a rate of 10 °C/min and were soaked at a temperature of 40 °C for 60 min, 80 °C for 60 min, and 200 °C for 480 min.

A 10-point Brunauer–Emmett–Teller (BET) method was used with a P/Po of 0.002, 0.005, 0.01, 0.015, 0.02, 0.025, 0.03, 0.035, 0.04, and 0.05. During adsorption, tolerance was 0.2, equilibrium was 60, timeout was 240, bath temperature was 77.35 K, bath thermal delay was 600 s, and backfill mode was helium. A 9 mm cell with a rod was used for all samples, and all samples were run using the fine powder mode with a pressure of 5 torr. The sample cell was massed before and after analysis before collecting the sample, as the surface area analysis is a non-destructive process. The cell was rinsed with water and sonicated with acetone before being dried in a 120 °C oven in between trials. A data report was generated using the BET demo template, and the t-plot method was used to estimate micropore volume of the sample.

### 3.3. Rhodamine B Photodegradation

Photodegradation experiments were conducted using Rhodamine B dye (RhB) as a model for photocatalysis. The photocatalytic reduction of RhB to colorless Leuco-RhB was monitored spectrophotometrically over time in the presence of each MOF powder. All photodegradation trials were conducted using a Persee Analytics T8DS UV Spectrometer (Persee Analytics) outfitted with a custom 3D-printed chamber that isolated samples from ambient light. The heterogeneous samples were prepared using 50 mL of 25 μM RhB in 5 mM acetic acid/sodium acetate buffer (pH = 4) and 15 mg MOF. The buffer ensures that RhB dye remains mostly in the cationic state, which gives strong fluorescence. In alkaline conditions, RhB converts to the non-fluorescent spirolactam, which is zwitterionic. Each heterogeneous sample was then left to be stirred in darkness for 1 h until physical adsorption equilibrium of RhB to MOF was achieved. After reaching adsorption equilibrium, white light was irradiated onto the sample chamber using a xenon lamp to simulate sunlight, and the disappearance of RhB was monitored by tracking the lambda max of RhB absorbance (~550 nm) over time. Before each measurement, 3.5 mL aliquots of heterogeneous solution were centrifuged for 5 min to separate solid MOF from RhB solution and the supernatant was collected for analysis.

### 3.4. Scavenging Experiments

Disodium ethylenediamine tetraacetate (EDTA-Na_2_) and ethanol were used as hole and electron scavengers, respectively, to elucidate the mechanism of photodegradation. Briefly, RhB photodegradation experiments were carried out using the same method described previously in Section 3.3 for MIL-125-NH_2_ 150 °C with the addition of each scavenger to solution. One experiment used 2 mM EDTA-Na_2_ as a hole scavenger and another experiment used 2 mM ethanol as an electron scavenger.

### 3.5. Crystallinity Retention Experiments

A heterogeneous solution containing 100 mg of MOF submerged in 50 mL of 25 μM RhB and 5 mM acetic acid/sodium acetate buffer (pH = 4) was left to be stirred in darkness for 1 h. After physical adsorption of RhB to MOF was achieved, the sample was irradiated with light while stirring for 24 h using a Xenon Series 9000 Model 9300 Xe lamp (Luxtec, Quebec, QC, Canada). After 24 h of irradiation, each sample was centrifuged for 30 min to separate solid MOF from RhB solution. The supernatant was discarded and the resulting MOF powder was washed in 10 mL of MeOH for 30 min, and then centrifuged again for 30 min before drying overnight under vacuum. The dry MOF powder was collected and analyzed by PXRD.

### 3.6. Physical Adsorption Kinetics

A calibration curve of absorbance vs. concentration for RhB at 10, 7.5, 5, 2.5 and 1 μM concentrations was first created for concentration determination. To evaluate physical adsorption of RhB dye to the MOF framework, a continuous flow analysis using a UV/Vis spectrometer was performed in the dark. Briefly, ~15 mg of MOF was added to a liquid column that recirculated 15 mL of 10 μM RhB solution through a 1 cm flow cell in the UV/Vis spectrometer and back into the column using a peristaltic pump. Experiments were run using MIL-125-NH_2_ 120 °C, MIL-125-NH_2_ 150 °C, pristine UiO-66-NH_2_ and UiO-66-NH_2_ 6.6% FA MOFs. Each MOF was added to the column after the first recording of RHB absorbance, and subsequent readings were taken every 5 min over a period of 1 h until adsorption equilibrium was established.

## 4. Conclusions

This study examined defect-engineered Zr(IV)- and Ti(IV)-based MOFs using acid modulators for UiO-66-NH_2_ and variable-temperature synthesis for MIL-125-NH_2_ to enhance photocatalytic performance. The presence of defect states was determined by monitoring changes in surface area, pore volume, IR spectra, bandgap, and XRD patterns. UiO-66-NH_2_ and MIL-125-NH_2_ frameworks were retained across synthesis temperatures and modulator concentrations, except for MIL-125-NH_2_ synthesized at 160 °C, which showed evidence of incomplete synthesis and partial framework formation. At 160 °C, the MIL-125-NH_2_ product became amorphous and showed sharp declines in surface area and bandgap, indicating poor crystallization.

Temperature modulation during synthesis in MIL-125-NH_2_ imparts large defects which eventually generate mesopores and cavitations, resulting in a slight decrease in surface area. These defects reach a critical size around 140 °C, and higher temperatures only generate more defects across the lattice. XRD shows that secondary phases become more prominent as the quantity of mesopores and cavitations increases.

Chemical modulation in UiO-66-NH_2_ is more “mild,” generating small defects spread throughout the MOF crystal with little change to the framework. Surface area and pore size depend heavily on the type of modulator used. FA is a stronger acid than AA, creating stronger competition for the organic linker that introduces metal node defects. It also readily diffuses into the MOF framework, modulating size and decreasing surface area. AA is a weaker acid and less likely to diffuse into the framework given its larger size. An increase in the concentration of AA during MOF synthesis increases surface area and pore volume of the product as a result of missing linker defects introduced during modulation.

Photocatalytic RhB degradation experiments showed that the most effective UiO-66-NH_2_ photocatalyst was the one treated with 6.6% formic acid, while the best MIL-125-NH_2_ variant was the one synthesized at 150 °C. The photocatalytic effect observed in UiO-66-NH_2_ is attributed to increased reactive sites from metal defects, whereas the photocatalysis observed in MIL-125-NH_2_ (150 °C) is driven by a decreased bandgap resulting from substantial metal node defects. The MIL-125-NH_2_ synthesized at 150 °C performed best at photocatalytically degrading RhB in solution, reaching over 60% dye degradation in 5 h and over 90% dye degradation after 15 h.

Scavenger experiments confirm a hole–electron mechanism, where photogenerated holes and electrons interact with components in solution to produce multiple ROS species that degrade RhB absorbed to the MOF framework. PXRD conducted before and after 24 h of photodegradation reveals that defective MIL MOFs are much more resilient than defective UiO MOFs, losing only ~5% crystallinity for MIL-125-NH_2_ 150 °C compared to ~20% loss in crystallinity for UiO-66-NH_2_ 6.6%FA. Kinetic studies reveal that both crystal features and defects play a role in physical adsorption of RhB dye to the MOFs in solution. MIL MOFs with temperature-induced defects show faster adsorption rates due to their macro-sized pores, but FA-modulated UiO MOFs have higher adsorption capacity for RhB due to larger surface areas. Overall, these results show how structural and electronic properties of UiO-66-NH_2_ and MIL-125-NH_2_ can be altered using different defect engineering strategies to enhance photocatalytic performance.

## Figures and Tables

**Table 1:** Bandgap energies for synthesized MOFs.

MOF	Bandgap (eV)
UiO-66-NH_2_	2.76
UiO-66-NH_2_ 3% AA	2.85
UiO-66-NH_2_ 6% AA	2.82
UiO-66-NH_2_ 9% AA	2.85
UiO-66-NH_2_ 2.2% FA	2.87
UiO-66-NH_2_ 4.4% FA	2.85
UiO-66-NH_2_ 6.6% FA	2.85
MIL-125-NH_2_ 100 °C	2.74
MIL-125-NH_2_ 120 °C	2.76
MIL-125-NH_2_ 130 °C	2.70
MIL-125-NH_2_ 140 °C	2.61
MIL-125-NH_2_ 150 °C	2.39
MIL-125-NH_2_ 160 °C	1.38

**Table 2:** Surface area and pore volumes of tested MOFs.

MOF	Surface Area (m^2^/g)	Pore Volume (cc/g)	Adsorption Capacity (µg RhB/mg MOF)
MIL-125-NH_2_ 120 °C	1584	0.58	1.12
MIL-125-NH_2_ 150 °C	623	0.38	0.46
Pristine UiO-66-NH_2_	1163	0.44	0.51
UiO-66-NH_2_ 6.6% FA	1055	0.41	0.81

## Data Availability

The original contributions presented in this study are included in the article/Supplementary Materials. Further inquiries can be directed to the corresponding author.

## Supplementary Materials

The following supporting information can be downloaded at https://www.mdpi.com/article/10.3390/molecules31071080/s1, Figure S1: SEM/EDS for Temperature Modulated MIL-125-NH_2_; Figure S2: SEM/EDS for FA/AA Modulated UiO-66-NH_2_; Figure S3: Band Gap Measurements for Defective UiO-66-NH_2_ MOFs; Figure S4: Band Gap Measurements for Defective MiL-125-NH_2_ MOFs; Figure S5: Comparison of defective UiO-66-NH_2_ modulated with acetic acid and formic acid photodegradation results; Figure S6: Comparison of defective MIL-125-NH_2_ photodegradation results; Table S1: Surface Area, Pore Volume, and Band Gap Measurements For All Tested MOFs; Table S2: R^2^ Values of Kinetic Models Fit for Dye Adsorption.

## Abbreviations

The following abbreviations are used in this manuscript:

AA	acetic acid
FA	formic acid
