# Supplementary material for: Defect Engineering in Zr (IV)- and Ti (IV)-Based Metal–Organic Frameworks to Enhance Photocatalytic Properties
Source: Molecules. 2026 Mar 25;31(7):1080. doi: 10.3390/molecules31071080 (PMC13074382; doi:10.3390/molecules31071080)
Supplement: Supplementary file 1 [file molecules-31-01080-s001.zip › molecules-4123717-supplementary.pdf]

Supplemental Data

Defect Engineering in Zr (IV)- and Ti (IV)- Based Metal-Organic Frameworks to Enhance Photocatalytic Properties

Adan Martinez, Emily Pearce, John Kurowski, and Daniel S. Kissel*

Lewis University, 2025

Table of contents

Figure S1: SEM/EDS for Temperature Modulated MIL-125-NH_2. . . . P3

Figure S2: SEM/EDS for FA/AA Modulated UiO-66-NH_2. . . . . P3

Figure S3: Band Gap Measurements for Defective UiO-66-NH_2 MOFs. . . P4

Figure S4: Band Gap Measurements for Defective MiL-125-NH_2 MOFs. . . P5

Figure S5: Comparison of Defective UiO-66-NH_2 Modulated with Acetic Acid and Formic Acid Photodegradation Results. P6

Figure S6: Comparison of defective MIL-125-NH_2 photodegradation results. . P7

Table S1: Surface Area, Pore Volume, and Band Gap Measurements For All Tested MOFs P7

Table S2: R^2 Values of Kinetic Models Fit for Dye Adsorption. . . . P8

Supplemental Figures

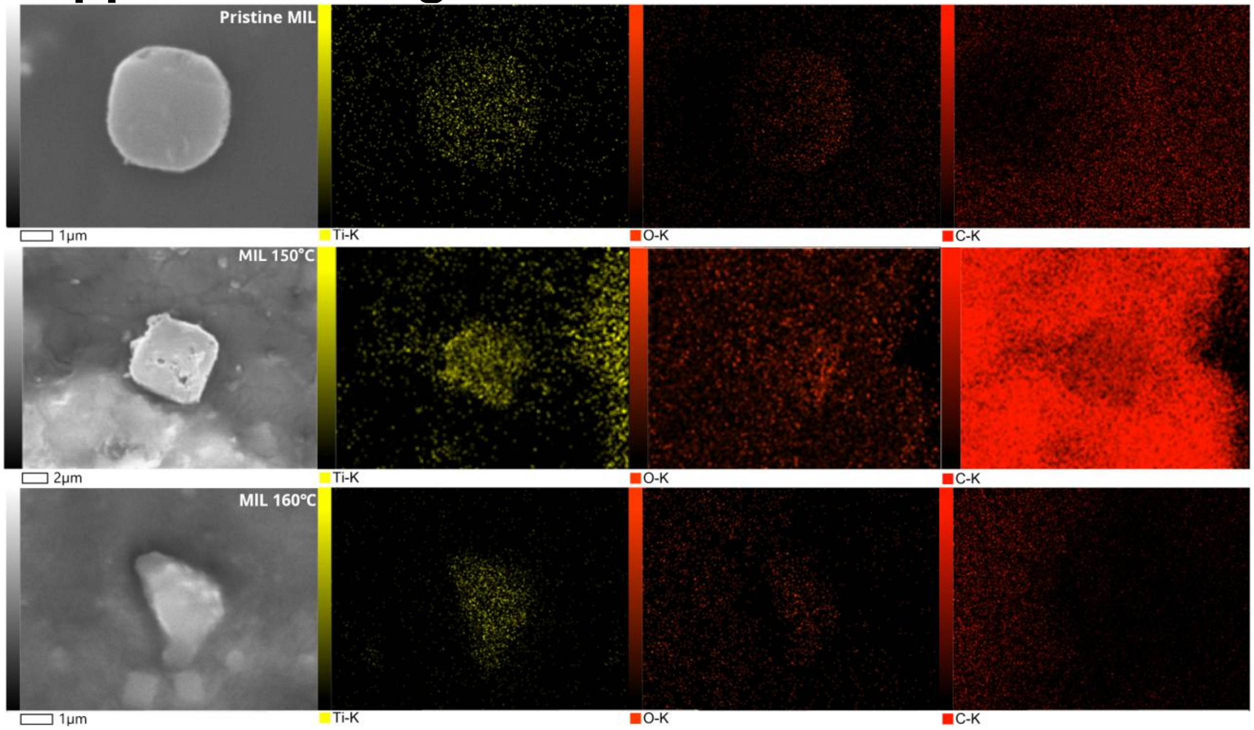

Figure S1: SEM/EDS for Temperature Modulated MIL-125-NH_2

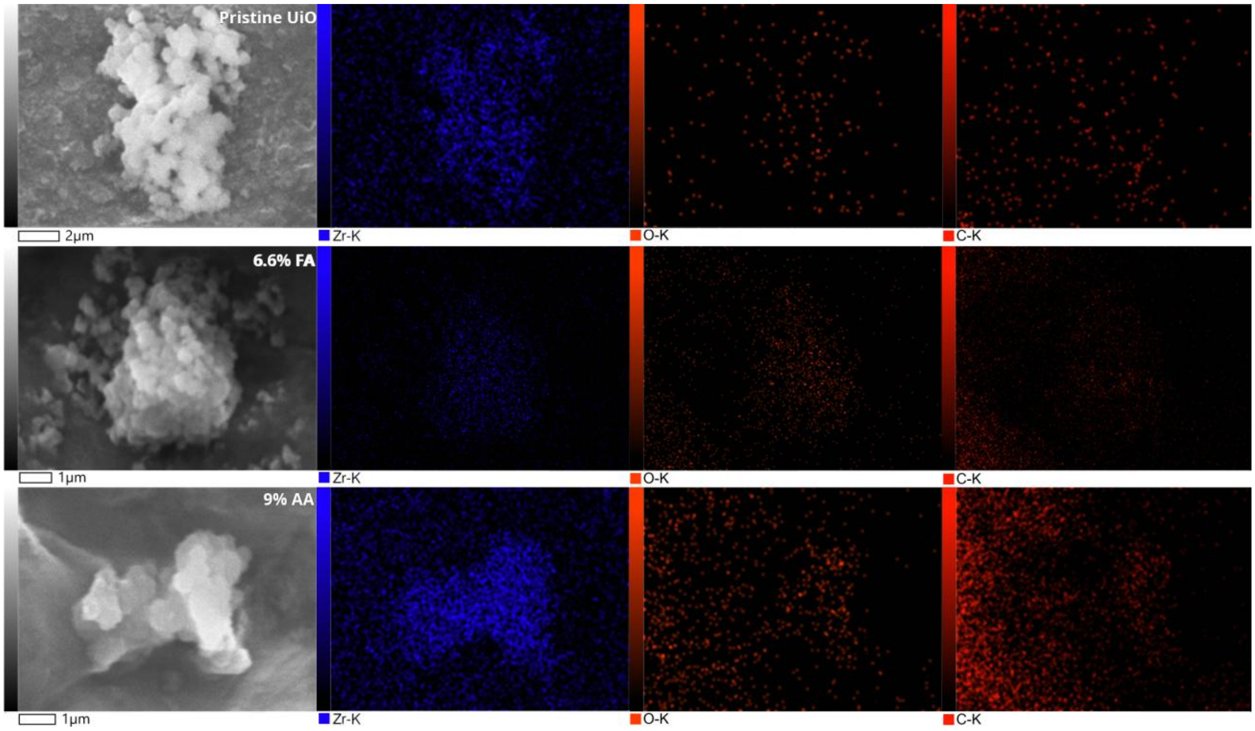

Figure S2: SEM/EDS for FA/AA Modulated UiO-66-NH_2

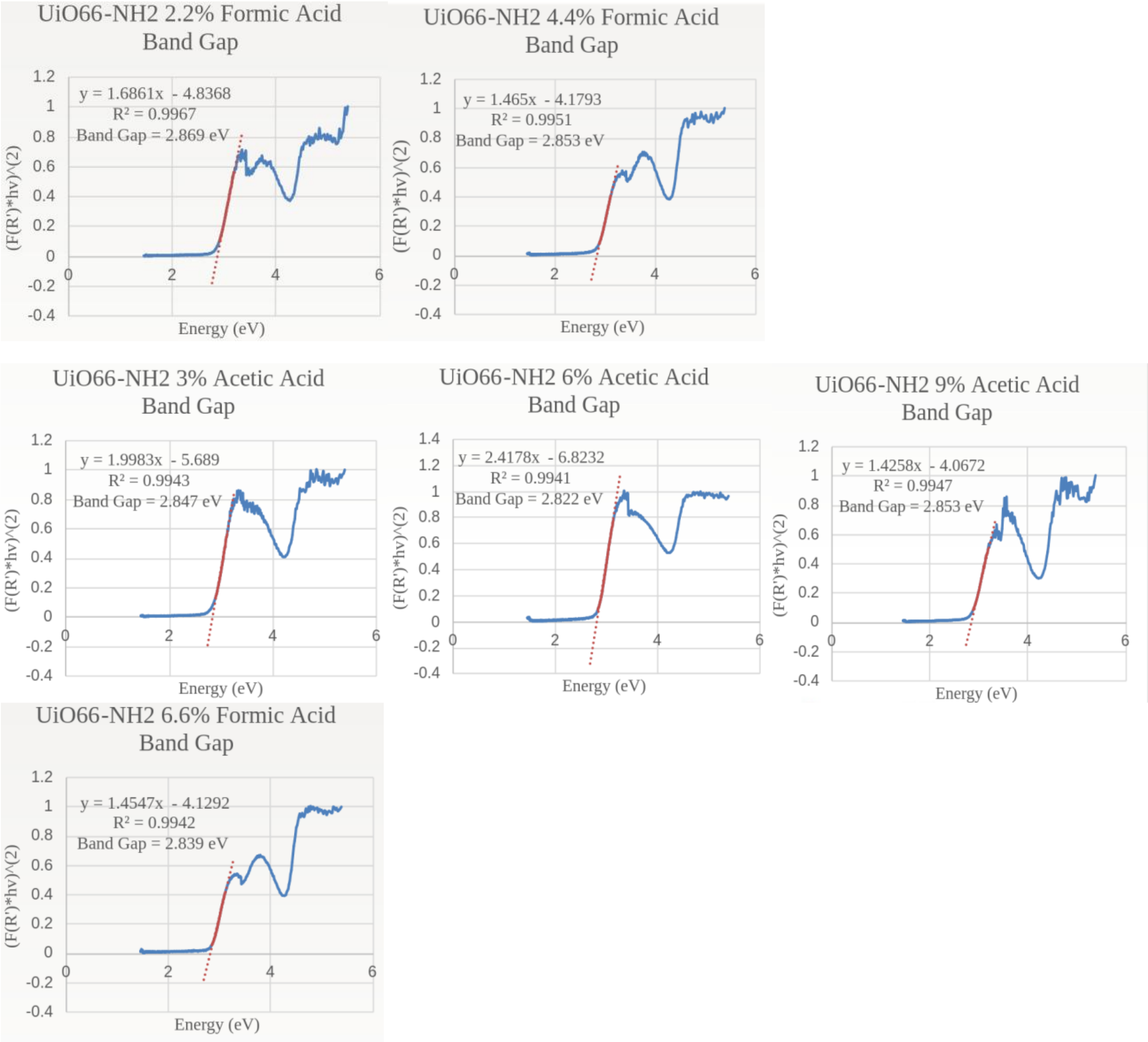

Figure S3: Band Gap Measurements for Defective UiO-66-NH_2 MOFs

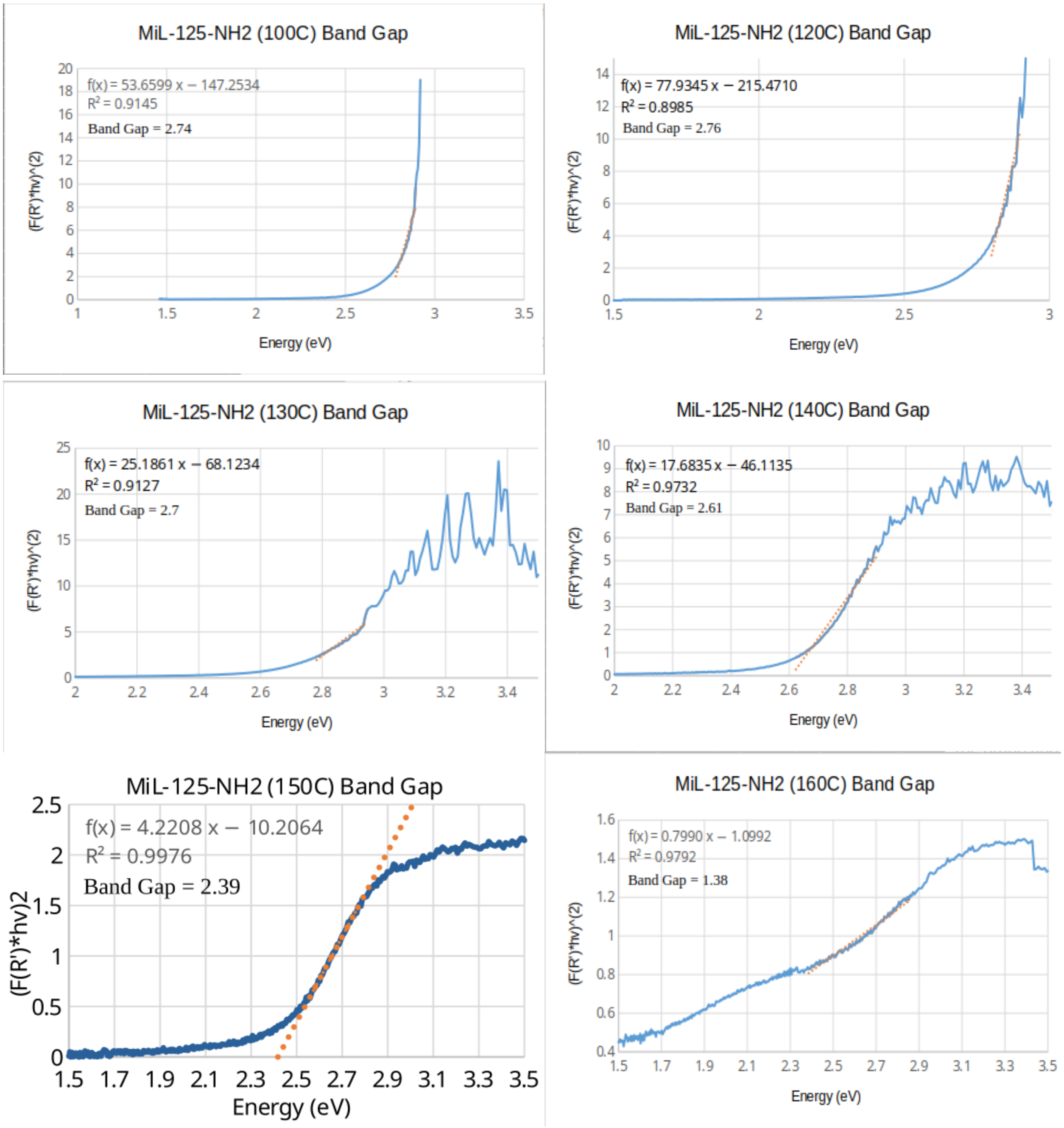

Figure S4: Band Gap Measurements for Defective MiL-125-NH_2 MOFs

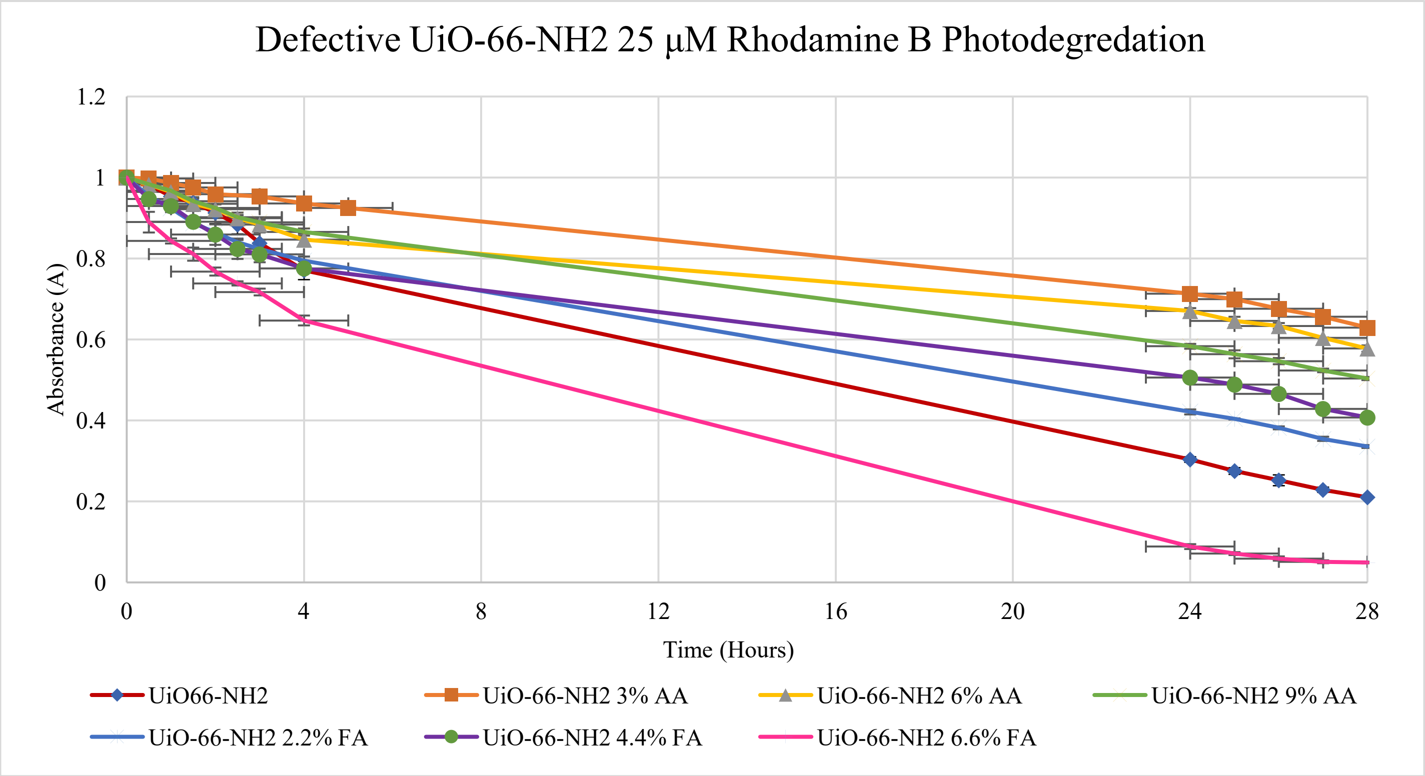

Figure S5: Comparison of defective UiO-66-NH_2 modulated with acetic acid and formic acid photodegradation results.

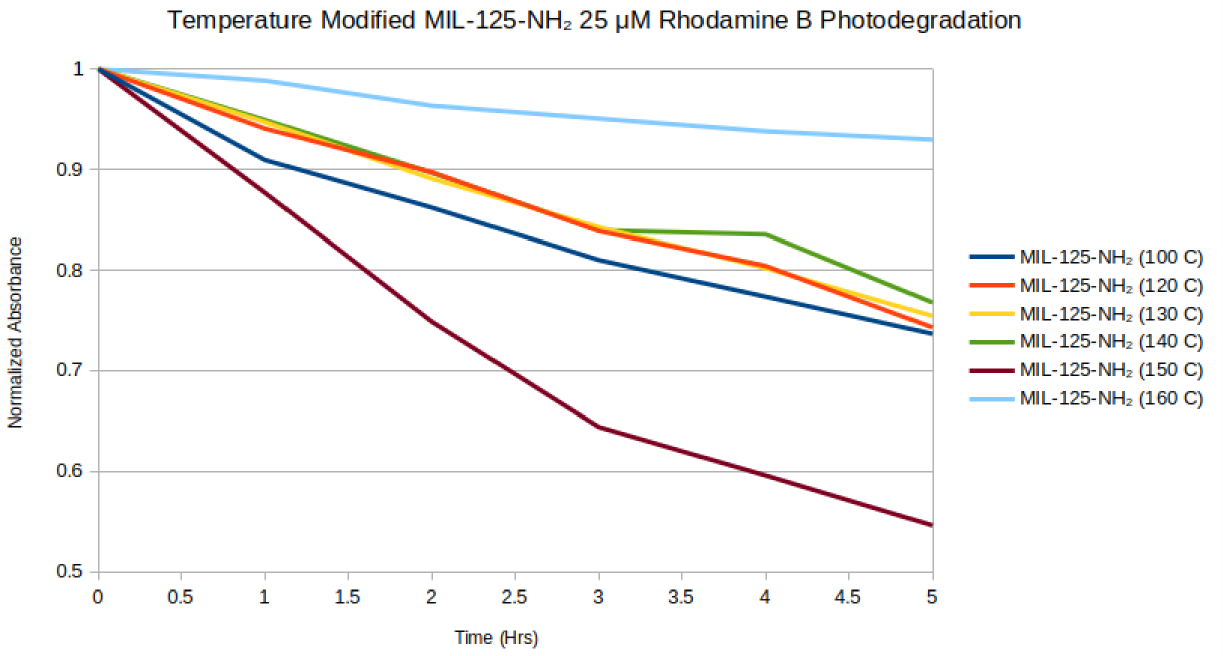

Figure S6: Comparison of defective MIL-125-NH_2 photodegradation results

Table S1: Surface Area, Pore Volume, and Band Gap Measurements For All Tested MOFs

MOF	Surface Area (m^2/g)	Pore Volume (cc/g)	Band Gap (eV)
UiO-66-NH_2	1163	0.4381	2.76
UiO-66-NH_2 3% AA	1095	0.389	2.85
UiO-66-NH_2 6% AA	1192	0.4283	2.82
UiO-66-NH_2 9% AA	1280	0.4717	2.85
UiO-66-NH_2 2.2% FA	1210	0.4465	2.87
UiO-66-NH_2 4.4% FA	1080	0.4104	2.85
UiO-66-NH_2 6.6% FA	1055	0.408	2.85
MIL-125-NH_2 (100 ℃)	1397	0.5151	2.74
MIL-125-NH_2 (120 ℃)	1584	0.5808	2.76
MIL-125-NH_2 (130 ℃)	1244	0.4744	2.7
MIL-125-NH_2 (140 ℃)	853	0.4027	2.61
MIL-125-NH_2 (150 ℃)	623	0.38	2.39
MIL-125-NH_2 (160 ℃)	172	0.1425	1.38

Table S2: R^2 Values of Kinetic Models Fit for Dye Adsorption.

MOF	First Order R^2	Second Order R^2	Pseudo First Order R^2	Pseudo Second Order R^2
UiO-66-NH_2	0.9777	0.9758	0.9777	0.6494
UiO-66-NH_2 6.6% FA	0.9687	0.9768	0.9793	0.5049
MIL-125-NH_2 (120 ℃)	0.917	0.9462	0.9428	0.1786
MIL-125-NH_2 (150 ℃)	0.915	0.9203	0.917	0.8746
